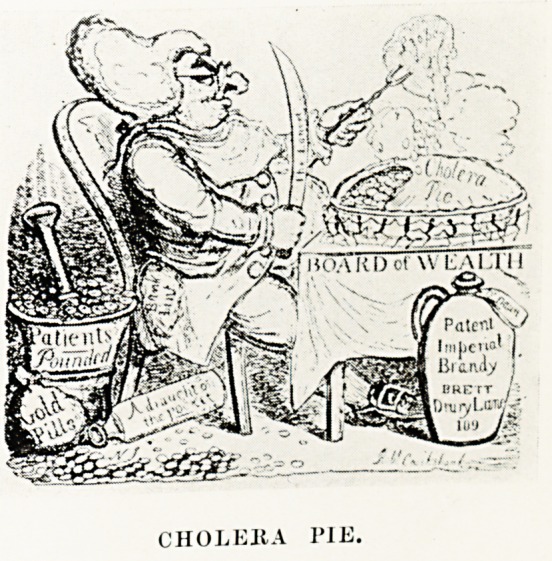# The Twenty-Ninth Long Fox Memorial Lecture: Popular Misconceptions in Medical Matters

**Published:** 1940

**Authors:** T. B. Davie

**Affiliations:** George Holt Professor of Pathology in the University of Liverpool


### The Bristol Medico-Chirurgical Journal

" *Scire est nescire, nisi id me*
*Scire alius sciret.*"

WINTER, 1940.

### THE TWENTY-NINTH LONG FOX MEMORIAL LECTURE:

BY

PROFESSOR T. B. DAVIE, M.D., F.R.C.P.,
*George Holt Professor of Pathology in the University of Liverpool,*

DELIVERED IN THE UNIVERSITY OF BRISTOL ON TUESDAY, 2nd JULY, 1940.

THE VICE-CHANCELLOR (Dr. T. LOVEDAY, M.A., LL.D.) *in the Chair.*

ON

### POPULAR MISCONCEPTIONS IN MEDICAL MATTERS.

In my search for a subject suitable for this address, I recalled that several of the more recent Long Fox Lectures were concerned with the medical lore and practices of ancient or primitive peoples. I have therefore been emboldened to attempt a brief survey of some of the commoner present-day misconceptions in medical matters extant among the general population of this country, and where possible to trace the origins of these mistaken notions, many of which bear the fossilized imprint of antiquity.

It is inevitable that misconceptions should arise among the uninitiated in relation to any specialized study, and in general such aberrant views are merely the outcome of simple ignorance. The more specialized and difficult the study, the more elementary or basic are the misconceptions likely to be, and the less liable are judgments or opinions based on them to affect harmfully the holders of such false concepts. On the other hand the nearer the specialist study approaches the daily life and experience of the general population the more significant become such misconceptions.

The study of medicine is a specialization, which in almost every aspect of its many activities impinges directly on the everyday life of the man in the street: and for this reason misconceptions are liable to exercise important influences on his welfare. It is precisely because the specialization of knowledge in the science and art of medicine in general does not take its secrets entirely beyond the scope of understanding of the lay public that misconceptions of its principles are so dangerous. Some of the ancillary branches of medicine such as radiology, radium therapy, pathology and bacteriology are very widely removed from immediate and constant contacts with the lay man, and his mistaken views on these branches of study seldom do any harm; but the general practitioner has constantly to wage war against prejudice and opposition based on misconceptions concerning even the elements of medical knowledge.

In choosing examples of these common misconceptions I have been guided partly by my own personal experience, but more by the desire to select those which illustrate the processes by which they have arisen. I have also deliberately tried to avoid exposing to the public gaze some of the more serious inaccuracies of medical knowledge which may be found among members of even such a gathering as this; for it is unfortunately true that the finer points of misconceptions in medical matters are rife even among the educated.

The first type of misconception which I wish to consider, as it provides us with an interesting study in analysis, comprises those having a definite element of truth on which has been super-imposed some perversion of reasoning or interpretation. Examples of these misconceptions are numerous and most of them are relatively harmless. The belief that the swallowing of orange or grape pips is the cause of appendicitis is widely held and almost completely erroneous; but it is obviously based on the fact that in diseased appendices are often found small seed-like faecal concretions. Another and equally harmless example is the wide-spread belief that the objectionable smells emanating from drains, stagnant pools, etc., are in themselves capable of conveying infections. Here the basis is obviously a combination of two observations: first, that infected tissues are often foul-smelling, and secondly, that certain infections are air-borne. This simple misconception illustrates well the type of faulty reasoning, manifested daily in every walk of life, by which apparently logical combinations of separate truths lead to faulty conclusions.

Another common and simple example of the misconceptions based on half-truths is the almost universal belief that sitting in draughts is fraught with danger of infections and ills of various sorts. This is so near the truth that action based on the faulty interpretation of this observation has quite a serious significance; since although it is true that a temporary lowering of vitality by chilling of part or all of the body, such as might result from exposure to draughts, may bring to light or localize a rheumatic fibrositis or even enable bacterial infections to gain a foot-hold, the faulty extension of this truth to the exclusion of fresh air from bedrooms, and more particularly from sick rooms, must have harmful instead of beneficial effects on the health of the nation.

Consider the belief that wounding of the soft web of skin between the thumb and index finger is liable to lead to tetanus. This is almost certainly based on the observation that any deep wound of the hand is liable to be contaminated with street or garden dirt and hence is prone to infection with tetanus spores. But for some obscure reason wounds in this very localized part of the hand have erroneously come to be regarded as particularly fraught with this danger. Not quite in the same class, yet readily intelligible, is the belief that the eating of beetroot is a preventive of anæmia. On the other hand the popular saying that the eating of fish is good for the brain has little to justify it beyond the fact that brain workers, like others, benefit from digestible meals, though the similarity of the white colour of cooked fish and of brain tissue has probably lent support to this belief.

Misconceptions of this simple type in which the element of the half-truth is evident are very widely fostered by the advertisements of proprietary medicines: which, on the principle that it pays to advertise, obtrude themselves daily on our notice from hoardings, through our letter boxes and in our popular press. You are all familiar with them. In many the inaccuracies are obvious even to the untrained, but in others the perversion of the truth is more subtle and elusive. The article advertised may be perfectly harmless and even worthy. The danger lies only in the unjustifiable confidence of the users of these articles in the efficacy of their action, a confidence engendered by the subtle promulgation of pseudo-scientific half-truths without any balancing exposition of the rest of the full truth as far as it is known to us. It is not my intention to discuss the ethics of this form of advertisement, nor am I in the least concerned with the value or otherwise of the advertised article; I wish merely to draw your attention to the process by which these simple misconceptions in matters affecting the health of the individual and the nation are originated and fostered.*

The second large group of misconceptions comprises those in which it is difficult to find the basis of truth, but which appear to be the heritage of the twentieth century from the mysticism and charlatanry of the past. Some of these are frankly accepted as baseless superstitions and are believed and

* This part of the lecture was illustrated by examples of well-known newspaper advertisements.

acted upon only by the very young or the completely illiterate: for example, the ritualistic ceremonies by which children make believe to remove warts.

Though these childish rituals are hardly worthy of being considered as popular misconceptions they provide a link with others, more widely held by adults, which expose just as surely a persistent faith in the occult. A peculiar evidence of this is the unreasoning preference for coloured doctors exhibited by many of the inhabitants of the poorer parts of most of our cities. It need hardly be added that this is practically never taken advantage of by these doctors, and such of them as I have spoken to regret its implications while being amused at its origin. But there are other evidences of belief in the mystical elements in medicine. While charms, scarabs and lucky tokens are regarded by most men merely as childish but harmless indulgences, there are, nevertheless, large numbers of people who daily carry with them some charm not merely "for luck" but specifically to ward off some ill or ailment. These sops to the powers of darkness are often indulged in surreptiously and even shamefacedly, in the full recognition that intelligence and reason are being flouted. The wearing of a ring of plaited elephant's hair as a preventive against rheumatism is one of the commonest.

It is to be noted that the use of these charms is particularly indulged in connection with common diseases, and more especially for those ailments which are known or believed to be beyond the control of orthodox medical skill. It is as though the medical failure is recognized and regarded as justifying the recourse to other, supernatural, sources of help. Rheumatic ailments are peculiarly subject to this mystical form of treatment and many of you must know of some of the commoner charms in use. I personally know a man, brother of a dentist, who always carries a magnet in his trouser pocket to ward off lumbago; and a near relative of mine, a victim of rheumatoid arthritis, was recently urged by two fellow members of her book club to try sewing a piece of raw potato into her clothing.

From this it is an easy step to the wearing of lockets containing various chemical or other medicaments, claimed by their vendors to possess the power of fending off a variety of ills from chilblains to diabetes. Many of these magical lockets, necklaces or belts are sufficiently widely believed in, bought and used, to make it a paying proposition for their manufacturers to expend weekly and even daily enormous sums on large scale advertisements in various newspapers and periodicals.

Numerous other examples could be quoted to illustrate misconceptions in which the elements of mysticism and the power of charms are evident. The belief that a stye on the eyelid can be cured by rubbing it with a wedding ring is based partly on the mystic healing power of the king of metals and partly on the magic powers of the ring or circle.*

Another common but erroneous belief is that all our tissues are changed every seven years. In this there is the element of truth: our skin and mucous membranes which are subjected to the attrition of daily wear and tear are continually replaced; but there is a complete absence of knowledge of the fact that certain cells of our body, notably our brain cells, are incapable of regeneration. The mystic element resides in the choice of the magical number "seven." In connection with this magical power of numbers it has been suggested that the almost invariable instruction of doctors to their patients to take their medicine *thrice* daily is another legacy from the days of witchcraft and magic.

Probably the most widespread of all medical misconceptions is the belief that in some mysterious way drugs are an essential part of all good treatment. Pills, powders or bottles of medicine are practically demanded by patients. It is as though these drugs are regarded as some form of internal amulet or charm, and the periodical nauseation, griping or other discomfort associated with their taking serve as laudable reminders to the patients that they are getting well. This blind faith in the value of drugs is by no means confined to the ignorant. It is recorded that Thomas Carlyle, hearing of the illness of his friend Henry Taylor, visited him bringing what was left of a bottle of medicine which had benefited Mrs. Carlyle in one of her illnesses.

The search of the alchemists of the middle ages for the elixir of life and for the perfect panacea is still in evidence though under new guise. You will remember the exposure by a well-known weekly periodical of the scandal of a much advertised product some years ago. In that particular case the debunking was based on chemical analysis which revealed the complete absence from the "panacea" of any constituent capable of possessing medicinal properties. To-day the exploitation of the credulity of the public is somewhat more subtle, as the majority of the much advertised and widely used proprietary medicines have definite medicinal value; and while a small proportion of the advertisements may err on the side of exaggeration in respect of the curative and prophylactic properties possessed by the article advertised, the real abuse is attributable to the public itself and is based upon a very common and easily intelligible popular misconception. This consists first of mistaking the symptom for the disease and secondly of assuming that a particular symptom is always evidence of the same disease. In its simplest form this misconception is the basis of the almost universal use of certain household remedies such as castor oil for abdominal pain and aspirin for headache. The pathological conditions causing abdominal

* Is not the *wedding* ring used because it has been blessed by the priest? —Editor.

pain are numerous, and very few indeed are really benefited by the hurried evacuation of the contents of the alimentary canal; the analgesic properties of aspirin can have permanent beneficial effects in only a small proportion of the disease states productive of headaches; but these facts never really affect the reasoning of the sufferer. When, therefore, in the newspaper, which he has chosen for its political colour and not for the standing of the writer of its medical column, our man in the street reads in large print, to the accompaniment of more or less convincing illustrations and personal testimonials, that somebody's purple pills will cure the aches, pains and stiffnesses of joints, he lays in a stock of these; and thereafter administers them to each and every member of the family—to himself for the gout he inherited and has aggravated by an over-indulgence in alcohol; to his wife for her prepatellar bursitis or "housemaid's knee"; to his son for the ankle wrenched at football; to his young daughter for the swollen, painful elbow joint of acute rheumatic fever; to the baby of the family for the tender shoulder of scurvy, and to his aged parents for their creaking joints of osteo-arthritis.

To be fair to our man in the street it should be pointed out that not only is every patient concerned primarily with the pain and discomfort of his symptoms rather than with the actual underlying disease, but also that the medical profession recognizes this fully and regards it as its solemn responsibility to provide symptomatic treatment for the alleviation of suffering: even where the ideal of simultaneous eradication of the basic pathologic changes cannot be attained. Mistaking the symptom for the disease is thus almost inevitable and becomes dangerous only when the obstinate persistence in the use of proprietary or household symptomatic remedies postpones medical investigation of serious diseases.

Closely allied to these misconceptions are some which result from other well recognized processes of faulty reasoning, such as those based on the *post hoc ergo propter hoc* conclusions, and others arising from the apparently irresistible tendency to generalize from a particular. In the absence of close critical analysis most people believe that because they have subjected themselves to treatment of a particular type and have subsequently enjoyed a general improvement in health, therefore *that treatment* was the cause of their recovery. This line of reasoning largely accounts for the popularity of spas and health centres, and is undoubtedly exploited at some time or another by most medical practitioners. There are literally thousands of people who swear by their favourite spa or health centre. In one case it is the drinking of some waters—usually more or less unpleasant, or even nauseating; in another it is some form of bath—radio-active, chalybeate, sulphur or mud. Again it may consist in the wholesale consumption of a particular article of diet—wholemeal bread, grapes or oranges; or it may depend upon the avoidance of some similar simple article of diet. In all these cases the patient fails to realize that the beneficial results of his course of treatment probably arise from the regime enforced, the regularity and reduced quantity of his meals, or the enforced hours of rest and recreation.

Faith in these stunt treatments is by no means a product of the twentieth century. Howard H. Haggard, in his *Devils, Drugs and Doctors*, refers to these and to faith-healing cults as "revivals of primitive, early Christian, and mediæval practices." He draws attention to the fact that they are all more or less short-lived. The bizarre claims and daringly unorthodox tenets which "impress one generation become merely amusing in the greater sophistication of the next." The seventeenth-century sale of beds which provided painless childbirth or of chairs which prevented rheumatism, was succeeded in the eighteenth century by the "celestial beds," which assured immediate fertility to childless couples. These were duly succeeded in the nineteenth century by treatments which guaranteed longevity; and they in turn have been dismissed with something like scorn, though our B.B.C. still occasionally brings to the microphone some centenarian to tell us the secret of his hundred years. To-day the stunts are less crude and, as previously indicated, have in most cases at least, a semi-scientific basis. It is, however, noteworthy that the modern promulgators appear to be no more able than were their predecessors (*e.g.* James Graham who developed a plan to promote longevity) to profit personally by their schemes.

The popularity of these stunt cures has many affinities with the vogue of faith-healing. It is interesting to note that cure by the laying on of hands, originally the prerogative of the church, passed in the course of centuries by way of the king through his nobles to the common people. The classical example of this is "touching for the King's evil" or scrofula, which is supposed to have started with Edward the Confessor. To-day some of these faith-healing cults have so many disciples that it would be indiscreet to do more than draw attention to the belief which, in one way or another, underlies them all: that orthodoxy is inherently narrow, and that any large professional or artisan class following an orthodox calling inevitably considers its own interests to the detriment of the population it serves. A corollary to this interpretation is, that any opposition by such a closed corporation against those who break away from its tenets is founded on a jealous fear that such unorthodox views will jeopardize the easy tenor of a comfortable and remunerative routine. From this it is an easy step to the assumption, which partakes of a highly satisfying discovery, that the unorthodox view is more probably correct than the orthodox. Oscar Wilde embodied this in one of his conceited epigrams: "When people agree with me, I know I am wrong."

This misconception is not confined to medical matters: there is hardly a profession against which the accusation of suppression of originality is not levelled, and history is rendered the more interesting by the records of the struggles and eventual victories of the great rebels and revolutionaries. In medicine the names of Pasteur, Lister and Jenner are quoted in support of those who distrust all orthodoxy, and in modern times stage, screen and popular press have greatly fostered these ideas. In such matters it is usually forgotten that any wrong done by orthodoxy in opposing these great originators of new thought and knowledge represents but a small debit balance against the real value of its incessant fight against quacks and charlatans. Opposition to new advances in medical thought is also not the prerogative of the die-hards of the profession. Servetus was burned at the stake for expounding his views, *inter alia*, on the pulmonary circulation ; Jenner's discoveries were ridiculed by the caricaturists, and to-day opposition to medical treatment of one form or another is exalted by thousands to the level of religious fanaticism.

Misconceptions as to the guiding principles animating the orthodox medical corporations are in many minds bound up with the seldom-voiced but often sensed accusation that the art of healing is prostituted to the financial cupidity of its practitioners. The day may be past when the physicians of the land are depicted as rejoicing in the profits accruing from the ravages of epidemics (as caricatured by Cruikshank in his *Cholera Pie*), but this suspicion is still undoubtedly more prevalent than deserved. A mild-natured friend of mine, after visiting an aged and wealthy acquaintance who was slowly dying of cancer, was so affected by his over-sentimental recollections of the old man's sufferings that in an unguarded moment he expressed the view that it was a shame that merely because the man was rich his medical attendants were keeping him alive, whereas if he had been poor they would long since have allowed him to pass out of his misery. The doctor is only too subject to human frailties, but every one who has worked with him in the course of his training and in his hospital and general practice will agree that this accusation is seldom deserved. The very subterranean nature of the antipathy engendered by this misconception as to the relationship between the doctor and his patient is the more regrettable as it becomes so readily linked with wider and largely political conflicts. Not only is the accusation exploited by the antagonists to the present system of medical practice in this country, but through this it becomes part of the great conflict between bureaucratic regimentation of all public and social services on the one hand and individualistic liberty of action on the other.

Perhaps because of the distrust of the " trade unionism " of medicine and the suspected money-grabbing proclivities of its practitioners, there has always been manifest another widespread

### PLATE II

ANCIENT METHODS OF HASTENING LABOUR.

DEATH AND THE BRIDE.

JENNER APPLYING VACCINATION.

CHOLERA PIE.

misconception. This is the belief that the medical profession is completely ignorant of a multitude of really valuable medicinal agents and therapeutic measures known to various members of the lay public. On this is founded the popularity of carefully-guarded family remedies, the success of herbalists, bone-setters, cheiropractists, etc., and the phenomenal sales of such books as *Everyman his own Doctor*. It explains, too, the recourse, usually surreptitious, of even the leaders of politics and industry to these quacks, many of whom are strongly reminiscent of the "Jack of all trades" portrayed by Rowlandson (1823) in his caricature of the dentist. These indulgences of the middle and upper classes are generally harmless, but the danger inherent in the practice needs no labouring by me.

In conclusion, I wish to remind you of the numerous misconceptions attaching to the medical aspects of sex, of which the basis is the ignorance and spurious modesty which for centuries shrouded the exact truths of sexual development, conception and parturition. The twentieth century has gone a long way towards dispelling the veil of mysticism and the murk of false teaching which characterized the medical practice of earlier times; but the legacy of prudery and hypocrisy engendered in the lay mind during the earlier times still manifests itself in unexpected quarters. It is true that the youth of to-day has few illusions in these matters, and many a parent has been staggered by the knowledge of his adolescent offspring to whom he has ventured to address a few words on "the facts of life"; but every medical man can testify to the tragedies which often result from completely unnecessary ignorance. The prudery of the seventeenth century, which demanded that the male doctor had to perform the manipulations of his confinement under the cover of a sheet in order to spare the blushes of his patient, has been replaced by an absence of self-consciousness in relation to the female figure which, among other things, has produced nudist colonies and a degree of realism in quasi-medical advertisements which is sometimes disturbing if not alarming. But on the whole misconceptions based on this type of prudery are now seldom encountered and rarely harmful. In the realm of obstetrics, however, the early Greek and Roman practices of hastening and encouraging labour by subjecting the parturient woman to rough handling of one kind or another are still fairly widely indulged in, though not in quite such a systematic manner, with results which are often undesirable or even disastrous.

It is probably in relation to venereal disease that some of the most lamentable misconceptions still exist among the poorer strata of our society. The advance of public health and social services have fortunately taken us far beyond the tragic times of the early sixteenth century, when syphilis spread through Europe to such an extent that Hans Holbein was led to depict gruesome Death jubilant as he accompanies the newly-married young woman to the bridal chamber. But venereal disease is still sufficiently prevalent to call for an intensive effort to eradicate the numerous misconceptions which continue to foster its spread. I would remind you of only one of these in which the influence of witch-craft is evident and, in particular, that phase of it known as the "transference of the curse." There is a belief in many parts of the country that a man can most easily rid himself of gonorrhœa by having intercourse with a virgin. Unpleasant as are the general implications of this belief, its actual application is most tragic in that it is a common cause of indecent assault on very young girls.

With these few examples I shall leave this subject of common misconceptions in medical matters. It is obvious that there is nothing unusual in their types as compared with misconceptions in other sciences and arts. Probably the only distinction which medicine can claim in this respect is the very large number of mistaken notions which beset it, and I have attempted to find the reasons for their multiplicity. For many of them the medical profession itself is responsible ; the lay public believing to-day what the doctors taught yesterday or the day before. It is obvious, too, that while most of these misconceptions are of no real importance, some are definitely harmful, and a few seriously dangerous. For all of them the cure is obviously education, not only in elementary hygiene, dietetics and simple human physiology, but also in the basic relationship of the doctor to his patient.

The illustrations in this article are taken from the book *Devils, Drugs and Doctors* by H. W. Haggard, published by William Heinemann (Medical Books) Ltd., and now available in a cheap edition at 12s. 6d. net.

## Figures and Tables

**Figure f1:**
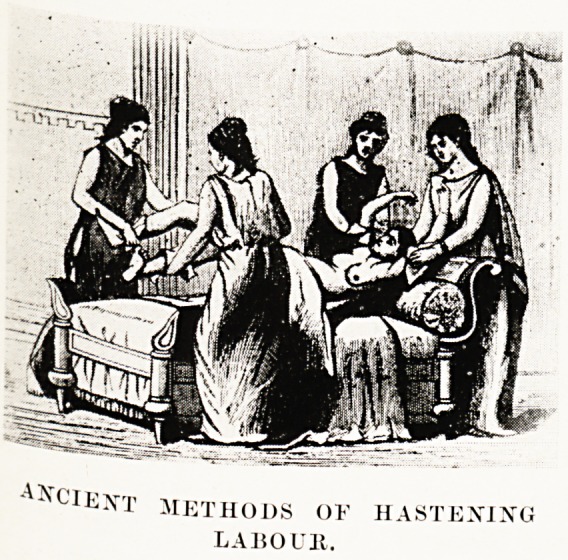


**Figure f2:**
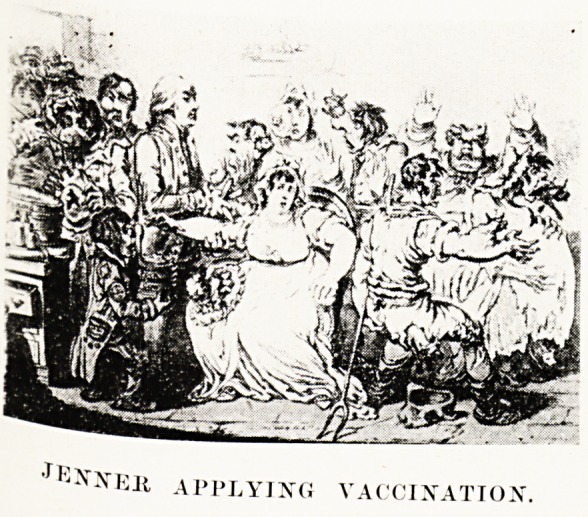


**Figure f3:**
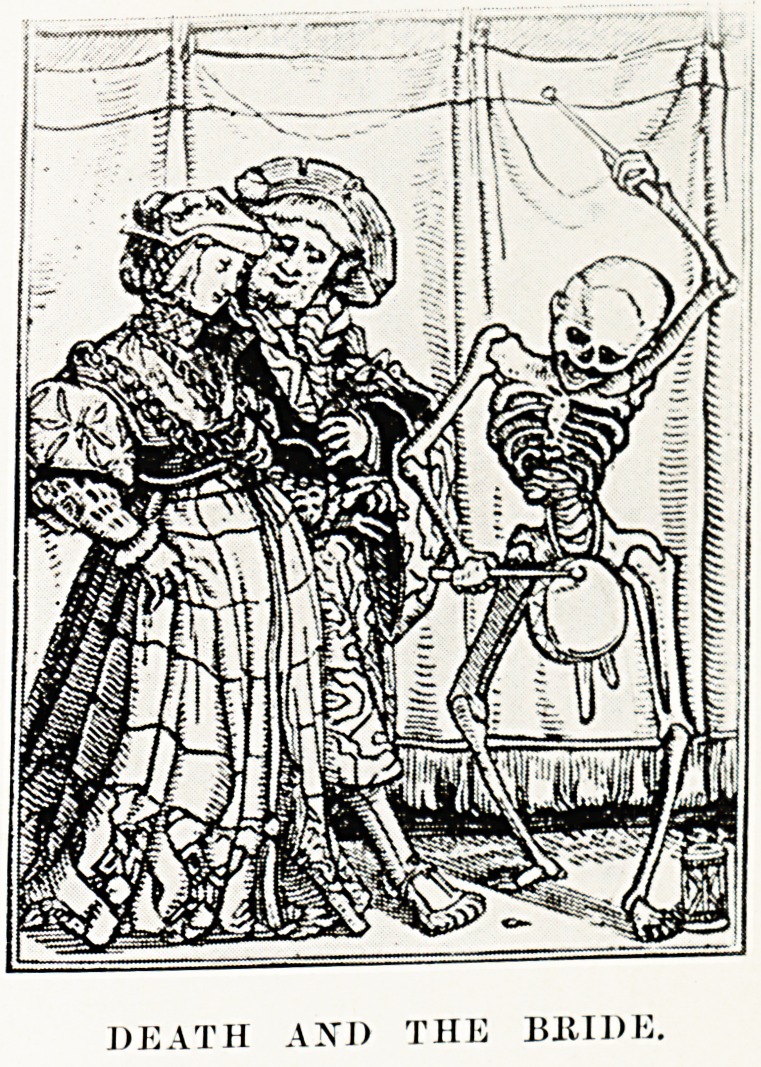


**Figure f4:**